# Physical Features of Visual Images Affect Macaque Monkey’s Preference for These Images

**DOI:** 10.3389/fnbeh.2016.00212

**Published:** 2016-11-02

**Authors:** Shintaro Funahashi

**Affiliations:** ^1^Kokoro Research Center, Kyoto UniversityKyoto, Japan; ^2^Department of Cognitive and Behavioral Sciences, Graduate School of Human and Environmental Studies, Kyoto UniversityKyoto, Japan

**Keywords:** preference, visual stimuli, choice behavior, eye movement, reward

## Abstract

Animals exhibit different degrees of preference toward various visual stimuli. In addition, it has been shown that strongly preferred stimuli can often act as a reward. The aim of the present study was to determine what features determine the strength of the preference for visual stimuli in order to examine neural mechanisms of preference judgment. We used 50 color photographs obtained from the Flickr Material Database (FMD) as original stimuli. Four macaque monkeys performed a simple choice task, in which two stimuli selected randomly from among the 50 stimuli were simultaneously presented on a monitor and monkeys were required to choose either stimulus by eye movements. We considered that the monkeys preferred the chosen stimulus if it continued to look at the stimulus for an additional 6 s and calculated a choice ratio for each stimulus. Each monkey exhibited a different choice ratio for each of the original 50 stimuli. They tended to select clear, colorful and in-focus stimuli. Complexity and clarity were stronger determinants of preference than colorfulness. Images that included greater amounts of spatial frequency components were selected more frequently. These results indicate that particular physical features of the stimulus can affect the strength of a monkey’s preference and that the complexity, clarity and colorfulness of the stimulus are important determinants of this preference. Neurophysiological studies would be needed to examine whether these features of visual stimuli produce more activation in neurons that participate in this preference judgment.

## Introduction

When humans and non-human primates observe a variety of visual stimuli, they often exhibit different degrees of preference toward these stimuli. The differences in preference for stimuli often influences choice behavior, such that visually preferred stimuli are behaviorally selected more frequently than others. Behavioral studies using capuchin monkeys and squirrel monkeys have revealed that they more frequently select stimuli with symmetrical and regular patterns compared to stimuli with unsymmetrical and irregular patterns (Anderson et al., 2005). Butler and Woolpy (1963) showed that rhesus monkeys preferred to see colored short movies compared with monochrome movies or photographs. Fujita (1987) presented a variety of images and photographs to five species of macaque monkeys (*Macaca fuscata, Macaca mulatta, Macaca radiata, Macaca nemestrina, Macaca arctoides*) and found that monkeys preferred watching photographs of the same species. Swartz and Rosenblum (1980) obtained results in bonnet monkeys similar to those obtained by Fujita (1987), and also showed that preferred stimuli could be used as a reward for monkeys to continue performing an operant conditioning task. Deaner et al. (2005) and Blatter and Schultz (2006) also showed that preferred visual stimuli could serve as rewards for rhesus monkeys to continue to perform behavioral tasks. Blatter and Schultz (2006) indicated that rhesus monkeys exhibit a preference for particular visual stimuli even though these stimuli are not directly related to reward and that the presentation of such preferred visual stimuli motivates the animals to choose these stimuli and can sometimes act as a reward.

Previously, Takebayashi and Funahashi (2009) used artificial fractal stimuli while rhesus monkeys performed a simple visual two-choice task and found that the monkeys exhibited different choice ratios for each of the stimuli. This difference in choice ratio could be due to the difference in the preference for these visual stimuli, such that more preferred visual stimuli would be more frequently selected than less preferred visual stimuli. However, it is not known whether the difference in the choice ratio among these stimuli depends on differences in the physical features of the stimuli (e.g., colorfulness, brightness, complexity and spatial frequency). It is also not known whether some physical features or parameters determine the choice ratio of a stimulus. Further, the difference in choice ratio might be affected by the difference in the materials that the stimuli show (e.g., fabric, paper, metal, plastic, glass and so on). In the present experiment, we used visual stimuli selected from the Flickr Material Database (FMD). Since this database includes pictures of a variety of materials (fabric, foliage, glass, leather, metal, paper, plastic, stone, water and wood), it should be ideal for examining whether the physical features of the materials photographed affect the difference in the choice ratio or the strength of the preference for stimuli. Since macaque monkeys we used had never seen these stimuli before the start of this experiment, we could exclude the influence of experience and memory regarding each stimulus. Further, previous behavioral studies with rather simple graphic patterns or motion stimuli showed that macaque monkeys preferred these stimuli (Anderson et al., 2005; Blatter and Schultz, 2006). The colorful and complex visual stimuli that we selected for the present study could be more attractive to these monkeys compared with the simple and monochrome visual stimuli used previously. Therefore, we expected that monkeys would exhibit a clearer preference for the stimuli we selected and that we could identify the parameters that determine the strength of the preference for visual stimuli more easily than in previous studies. These behavioral results are essential when we try to understand what neural mechanisms participate in preference judgment.

## Materials and Methods

### Subjects

Two rhesus monkeys (*Macaca mulatta*; Monkey K, 8.2 kg; Monkey M, 6.0 kg) and two Japanese monkeys (*Macaca fuscata*; Monkey H, 5.2 kg; Monkey Y, 5.1 kg) were used as subjects. Two rhesus monkeys had been used for other behavioral experiments examining spatial working memory. Two Japanese monkeys were used for neurophysiological experiments to examine orbitofrontal contribution in preference judgment of visual stimuli after finishing the present experiment. These monkeys were kept in individual home cages. Food was given *ad libitum*. Although water intake was not allowed in the home cage, the necessary amount of water (about 250–300 ml) was given as a task reward during the daily experiment. If necessary, additional water, fruit or vegetable was given in the home cage at the end of the daily experiment. Monkeys were given free water in the home cage during weekends. All experiments were performed under the guidelines issued by the Primate Research Institute of Kyoto University. The experiment was approved by the Animal Research Committee at the Graduate School of Human and Environmental Studies, Kyoto University.

### Apparatus

The monkeys were placed in a custom-made primate chair and performed behavioral tasks in a dark, sound-attenuated room. Visual stimuli were presented on a TV monitor (FlexScan 20TX, Nanao, Ishikawa, Japan), which was placed about 40 cm away from the monkey’s face. Before starting the experiment, we fixed a stainless steel device on the monkey’s skull to restrict its head movement during the experiment. This surgical procedure has been described in detail elsewhere (Takeda and Funahashi, 2002). We implanted an eye coil under the conjunctiva of either eye to monitor eye movements during the task (Judge et al., 1980). Monkeys’ eye movements were monitored using a magnetic search coil technique (Robinson, 1963). TEMPO software (Reflective Computing, Olympia, WA, USA) was used to control task events, select and present visual stimuli, monitor monkey’s eye movements and store behavioral data including eye movements.

### Visual Stimuli

**Figure 1:**
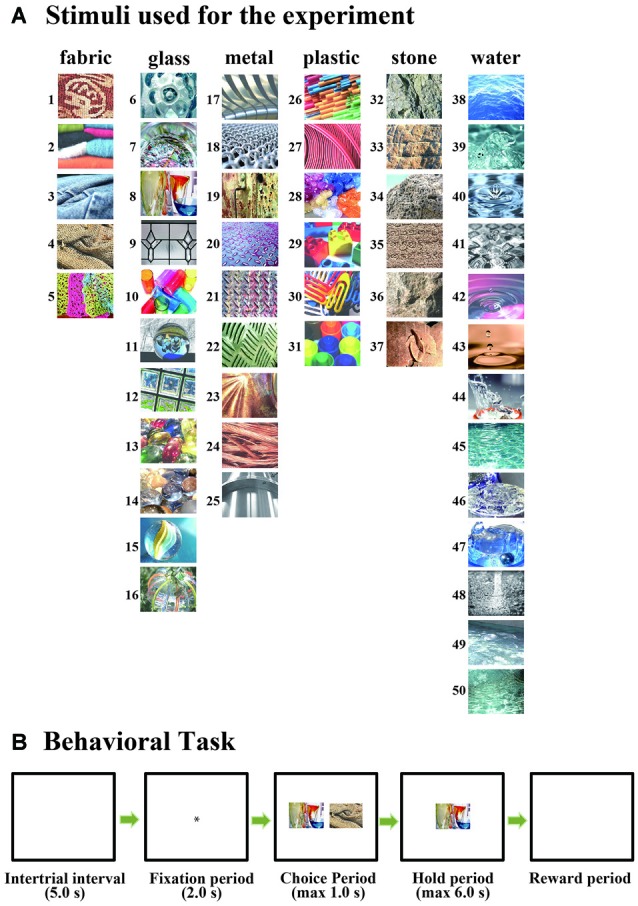
**(A)** Fifty stimuli selected from the Flickr Material Database (FMD) and used for the experiment. **(B)** The temporal sequence of the behavioral task. Monkeys performed a simple choice task in which they were required to choose one stimulus from among two stimuli presented simultaneously by their eye movements and then to continue to look at the selected stimulus for 3–6 s.

Visual stimuli used in the present experiment were obtained from the FMD^1^. We selected 50 pictures (5 of fabric, 11 of glass, 9 of metal, 6 of plastic, 6 of stone and 13 of water) from this database as original color stimuli (Figure 1A). We made two modified versions of each of these original 50 stimuli, black-and-white and colored-blurred versions, using Adobe Photoshop CS5 (Adobe Systems Inc., San Jose, CA, USA). For producing colored-blurred stimuli, we used original parameters of the “coarse” command in the “artistic expression” of the “filter” in Photoshop CS5. Thus, three versions of each of the 50 stimuli (original and two modified versions) were standardized to a visual angle of 10° × 10° using Adobe Photoshop CS5.

### Behavioral Task

Monkeys performed a simple choice task in which they were required to choose one stimulus from two simultaneously presented stimuli by their eye movements and then to continue to look at the selected stimulus for up to 6 s (Figure 1B; Greenberg, 1965; Takebayashi and Funahashi, 2009). After an inter-trial interval (ITI) of 5 s, a fixation target (small white circle) was presented at the center of the monitor. The monkey was required to continue gazing at the fixation target for 2 s (fixation period), and then two stimuli were presented simultaneously to the left and right of the fixation target (choice period). These two stimuli were selected randomly from among the 50 stimuli. The position where each stimulus was presented (left or right) was randomized over trials. During the choice period, the monkey was required to choose either stimulus by their eye movements. If the monkey looked at one stimulus for 1 s at least, we considered that the monkey had selected that stimulus. To confirm that the monkey made a correct choice, only the selected stimulus was presented at the center of the monitor immediately after the monkey’s selection, and the monkey was required to maintain looking at the selected stimulus for up to 6 s (hold period). If the monkey’s gaze remained on the stimulus throughout the entire hold period, we considered that the monkey had selected this stimulus, and a drop of water (0.3–0.5 ml) was given as a reward at the end of the hold period. If the monkey’s gaze left the stimulus during the hold period, the stimulus disappeared and the trial immediately terminated without a reward. The length of the hold period was randomized between 3 s and 6 s.

To examine whether or not the monkey’s selection of visual stimuli was affected by the physical properties of the stimuli, we also used black-and-white versions and colored-blurred versions of the original 50 stimuli to test their preferences as separate trial blocks after finishing behavioral tests using original sets of stimuli. We simultaneously presented a black-and-white version and an original color version of the same stimulus, or a colored-blurred version and an original version of the same stimulus, during the choice period. The monkeys were tasked to select either version of the stimulus by their eye movements. The positions where the two versions of the same stimulus were presented were randomized across trials. As in the original choice task, we considered that the monkey selected one version of the stimulus if it looked at that version for at least 1 s during the choice period and if it continued to look at this stimulus during the hold period. Either the black-and-white version or the colored-blurred version was introduced as a block of trials.

At the beginning of the present experiment, we tried to use the presentation of the visual stimuli themselves as a reward, since we hoped to exclude specific association effect between visual stimuli and reward. However, the monkeys’ performance rapidly deteriorated within a few sessions and, after they performed 10–20 trials, none of the monkeys performed the task any further without a liquid reward. Therefore, we used a liquid reward in this experiment to keep the monkeys’ motivation level constant during the experiment. The monkeys received the same amount (about 0.15 ml) of liquid reward, regardless of which stimulus was selected.

### Data Collection and Evaluation

Task information and behavioral data were collected by the TEMPO program and stored on magnetic media. Stored task information included the stimuli and their positions presented during the choice period, the stimulus selected in the choice period, the position of the selected stimulus (right or left), the time when each task event occurred, the time when the monkey started looking at the selected stimulus in the choice period, the time when the monkey started looking at the selected stimulus in the hold period, the time when the monkey broke fixation during the hold period, and horizontal and vertical eye movements. In the present study, a trial was defined as correct if the monkey continued to gaze at the selected stimulus until the end of the hold period and obtained a reward. We calculated the number of times that each stimulus was presented in the choice period, the number of times that each stimulus was selected in the choice period, and the number of times that the monkey obtained a reward when each stimulus was selected. Based on these values, we calculated the average percentage of trials when each stimulus was selected (choice ratio) and compared these values among stimuli within each stimulus set.

Using these choice ratios, we first examined the psychological similarity of the images by using a classical multidimensional scaling method (Torgerson, 1958; Mardia, 1978). We used the observed monkey’s choice ratio for each image under each pair condition in the choice task. For each subject, we first calculated the distance between arbitrarily selected images using the choice ratios of these images in trials in which these two images were presented during the choice period. We denoted this distance as the psychological distance between these two images. The distance between two images was defined as the difference in the animal’s choice ratio from 0.5 for that pair. For example, if the animal chose one image in a given pair of images in 15% of the total presentations and chose the other image in the other 85% of the total presentations, the distance between these two images was considered to be 0.35 (|0.15 − 0.5| = |0.85 − 0.5|). We created a distance matrix using all possible pairs from among the 50, and then applied a 2-D multi-dimensional scaling to this distance matrix.

The visual images used in this experiment exhibit a variety of physical properties; brightness, colorfulness and clarity. Therefore, some physical property of these visual images may have affected the monkeys’ selection. To examine this issue, we calculated average values of luminance, hue and saturation and the strength of the spatial frequency component for each image and examined correlations between the choice ratio and each of these physical parameters. For each image, we calculated the averaged luminance, hue and saturation from the RGB values. Luminance (*L*) was calculated as

(1) L = 0.299 * R+0.587 * G+0.114 * B

where *R*, *G*, and *B* denote the values of the red, green and blue channels, respectively. Hue is considered in terms the angular degree of the color in the color circle. Hue (*H*) is calculated as

(2) $$H\;=\;\text{arctan}\left(\sqrt{3}\left(G-B\right)/\left(2R-G-B\right)\right)$$

Saturation is calculated as the difference between the maximal and minimal values of RGB channels. In this study, luminance, hue and saturation were calculated for each pixel and then averaged within each image. For the calculation of the mean hue, we used directional averaging, where we averaged each of the Cartesian coordinates of the angles on the unit circle and then converted it to a polar coordinate. We also used the length of the averaged hue vector as an index of the variation of the hue in each image. The length of the averaged hue vector increases toward unity when the hues of the pixels in an image are similar, and decreases toward zero when they vary. Therefore, we subtracted the length of the averaged hue vector from one so that it would reflect the variation of the hues.

The strength of the spatial frequency component of each image was calculated based on the method proposed by Portilla and Simoncelli (2000). All color images were transferred into monochrome images using Photoshop. A steerable wavelet pyramid transform was applied to the luminance of each monochrome image to decompose the image into 12 sub-band images. These sub-band images include three scales (high, middle and low spatial frequencies) and four orientations (0°, 45°, 90° and 135°). With the use of these sub-band images, 12 sub-band statistics (log of mean magnitude of each sub-band) were obtained. Next, to examine whether or not spatial frequency components affected the monkey’s choice behavior, we calculated the correlation coefficient between the strength of the spatial frequency component and the choice ratio of each image.

## Results

### Database

**Figure 2:**
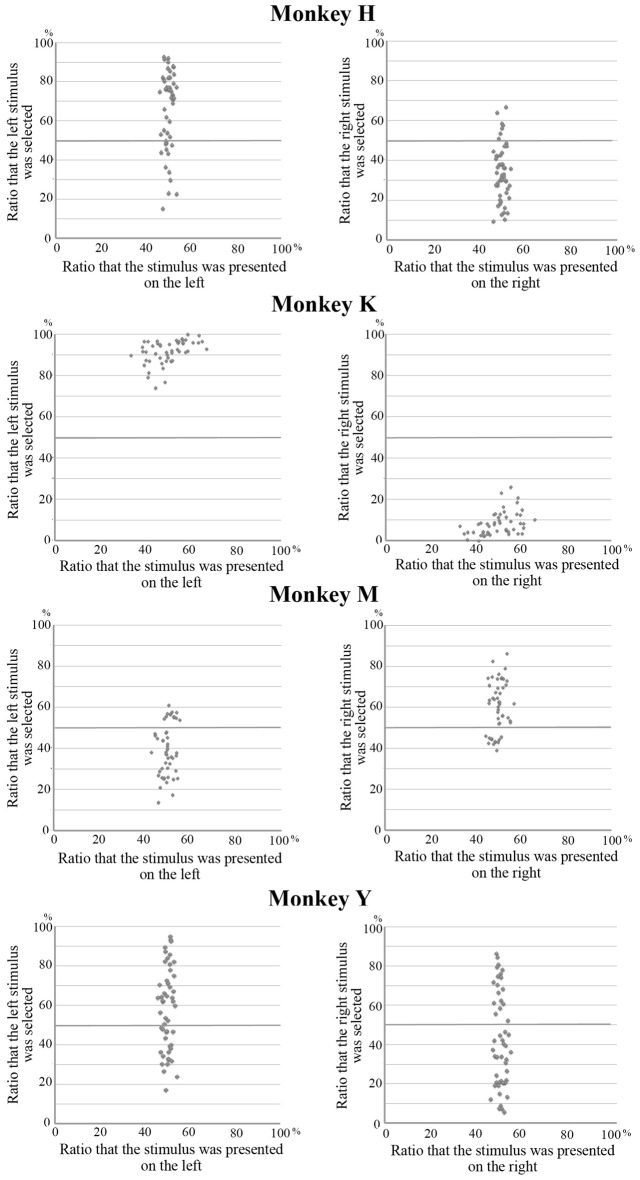
**Relation between the position of the selected stimulus and the position where this stimulus was presented.** The mean choice ratios for stimuli on the left or the right were 66% and 34% in Monkey H, 40% and 60% in Monkey M, and 58% and 42% in Monkey Y, respectively. Although these differences were statistically significant (paired *t*-test, *p* < 0.01), the overall choice ratio for each stimulus was significantly correlated with the choice ratio when the stimulus was presented on either the left or the right for these monkeys. However, in Monkey K, a strong spatial bias (92% vs. 8%) was observed in stimulus selection and no correlation was observed between the overall choice ratio for each stimulus and the choice ratio on the left and right.

The behavioral data in the present experiment are based on the results obtained from 48 sessions (a total of 18,445 trials) for Monkey H, 40 sessions (13,871 trials) for Monkey K, 40 sessions (9889 trials) for Monkey M, and 46 sessions (15,426 trials) for Monkey Y. In the present experiment, the monkeys needed to select one visual stimulus that was presented on either the right or the left during the choice period. They always received the same amount of liquid reward regardless of which stimulus they selected. Therefore, the monkeys might exhibit a spatial bias in their selections, such that they always selected the stimulus presented on one side regardless of which stimulus was presented. Figure 2 shows the relation between the position of the selected stimulus and the position where this stimulus was presented. The mean choice ratios for the selection of a stimulus on the left or the right were 66% and 34% in Monkey H, 40% and 60% in Monkey M, and 58% and 42% in Monkey Y, respectively. All of these differences were statistically significant (paired *t*-test, *p* < 0.01). However, the overall choice ratio for each stimulus was significantly correlated with the choice ratio when the stimulus was presented on either the left or the right for each of these monkeys (Monkey H, left, *r* = 0.905, right, *r* = 0.995; Monkey M, left, *r* = 0.880, right, *r* = −0.880; Monkey Y, left, *r* = 0.979, right, *r* = 0.985). However, in Monkey K, a strong spatial bias (92% vs. 8%) was observed in stimulus selection and no correlation was observed between the overall choice ratio and the choice ratio when the stimulus was presented on either the left or the right. These results indicate that, although all of the monkeys exhibited a spatial bias in stimulus selection where the strength of the bias varied among the monkeys, stimulus selection during the choice period depended on the preference for the stimuli in at least three monkeys (Monkeys H, M and Y).

### Selection Patterns of Stimuli

**Figure 3:**
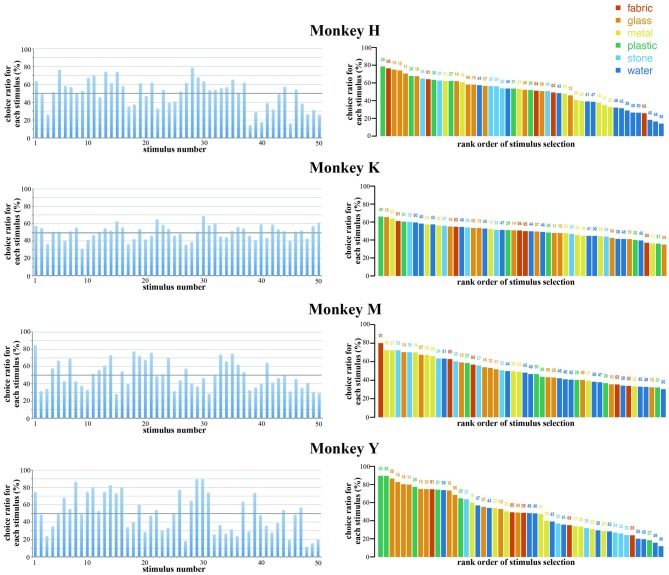
**Choice ratios of the 50 original stimuli in the four monkeys.** (Left) Choice ratios were arranged according to the stimulus number (see Figure 1A). (Right) Stimuli were arranged according to the rank order of their choice ratio. Five different colors indicate five different materials. Each monkey exhibited different patterns of choice ratios for the 50 original stimuli. The stimuli that exhibited higher choice ratios were not the same among the four monkeys. These results indicate that there are individual differences in the selection of visual images.

In the present study, monkeys were required to select one stimulus from two simultaneously presented visual stimuli by eye movements. The stimulus was considered to be selected by the monkey if the monkey looked at that stimulus for at least 1 s during the choice period and continued to gaze at that stimulus until the end of the hold period. We calculated the choice ratio of each stimulus based on the total number of trials that the particular stimulus was presented during the choice period and the total number of trials that this stimulus was selected and rewarded. Figure 3 shows the choice ratios of the 50 original stimuli in the four monkeys. Some stimuli were selected frequently while others were selected less frequently. The highest and lowest choice ratios were 79% (stimulus 28) and 14% (stimulus 38) in Monkey H (mean, 738 trials/stimulus), 68% (stimulus 30) and 31% (stimulus 9) in Monkey K (mean, 555 trials/stimulus), 84% (stimulus 1) and 28% (stimulus 15) in Monkey M (mean, 396 trials/stimulus), and 90% (stimulus 30) and 12% (stimulus 48) in Monkey Y (mean, 617 trials/stimulus). Each monkey exhibited different patterns of choice ratios for the 50 original stimuli. In the right panels of Figure 3, the 50 stimuli are arranged according to the rank order of their choice ratio. The stimuli that exhibited higher choice ratios were not the same among the four monkeys. The stimuli that were within the top five in rank order were 28, 5, 13, 15, 11 in Monkey H, 30, 22, 15, 50, 32 in Monkey K, 1, 18, 21, 35, 33 in Monkey M, and 30, 29, 8, 14, 16 in Monkey Y. These results indicate that there are individual differences in the selection of visual images.

In the present study, we used 50 visual images that included six different materials (fabric, glass, metal, plastic, stone and water) as stimuli. Comparison of the choice ratios among these materials revealed a statistically significant difference in three monkeys (Monkey H, *F*_(5,44)_ = 9.138, *p* < 0.01; Monkey M, *F*_(5,44)_ = 3.684, *p* < 0.01; Monkey Y, *F*_(5,44)_ = 7.615, *p* < 0.01). In Monkeys H and Y, *post hoc* comparisons (Bonferroni test) of the choice ratios among different materials revealed that the choice ratios of water and metal were significantly low, while the choice ratios of glass and plastic were significantly high compared with those of other materials. In Monkey M, the choice ratios of metal and stone were high compared with those of other materials. In Monkey K, no significant difference in the choice ratios was observed among the different materials. Again, the results showed that there are individual differences in the selection of visual images among monkeys.

### Selection Patterns of Stimuli were Maintained Across Sessions

**Figure 4:**
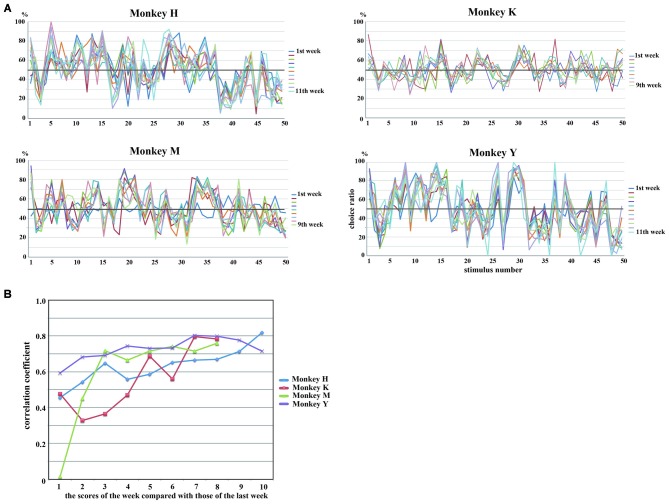
**(A)** Weekly changes in choice ratios of 50 stimuli across consecutive weeks. Lines of different colors indicate data obtained in different weeks. Although the choice ratio changed for some stimuli, the overall patterns of the weekly values of choice ratios for the 50 stimuli were maintained across consecutive weeks in all of the monkeys. **(B)** Temporal changes of correlation coefficients calculated between choice ratios of 50 stimuli obtained in each week and those obtained in the last week of the behavioral experiment. Statistically significant positive correlations (*p* < 0.01) were continuously observed across whole examined weeks in Monkeys H, K and Y and from the second week to the last week in Monkey M. Correlations gradually increased with time in all monkeys. Different colors indicate the results of different monkeys.

**Figure 5:**
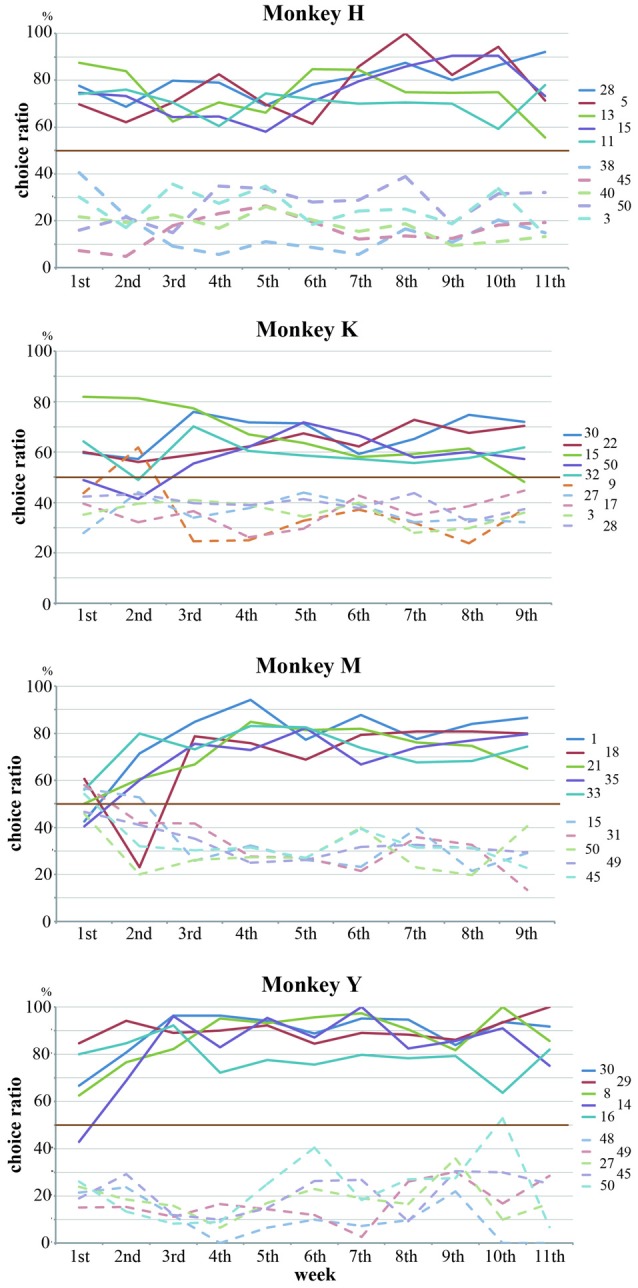
**Five stimuli with the highest choice ratios and five stimuli with the lowest choice ratios were selected for each monkey, and the temporal changes in the choice ratios for these stimuli were examined for 9 or 11 consecutive weeks.** Although the choice ratios for these stimuli fluctuated during the first or second week, each monkey tended to select some particular stimuli at higher choice ratios and other stimuli at lower choice ratios. Straight lines and dotted lines indicate the temporal changes for the stimuli with higher choice ratios and the stimuli with lower choice ratio, respectively. Different colors indicate the data of different stimuli.

The rank order of the choice ratios for 50 stimuli was maintained across experimental sessions for all of the monkeys. We collected behavioral data for 11 consecutive weeks in Monkeys H and Y and for nine consecutive weeks in Monkeys K and M. Figure 4A shows the weekly values of the choice ratios for the 50 stimuli across consecutive weeks. Although some stimuli showed a change in the choice ratio, the overall patterns of the weekly values of choice ratios for the 50 stimuli were maintained across consecutive weeks in all of the monkeys. To confirm the similarity of the overall patterns of choice ratios across weeks, we calculated correlation coefficients between choice ratios for each examined week and the choice ratios of the last week. As shown in Figure 4B, significant positive correlations (*p* < 0.01) were continuously observed across whole examined weeks in three monkeys (Monkeys H, K and Y) and from the second week to the last week in Monkey M. Correlation coefficients were gradually increased in all monkeys. Especially, the stimuli with higher choice ratios continued to have high choice ratios and those with lower choice ratios continued to have low choice ratios across consecutive weeks. To confirm these observations, we selected the five stimuli with the highest choice ratios and the five stimuli with the lowest choice ratios for each monkey and examined the temporal change in the choice ratios for these stimuli for 9 or 11 consecutive weeks. Figure 5 shows that, although the rank order of these stimuli fluctuated during the first or second week, the rank order eventually settled and was maintained during the remaining weeks. Thus, each monkey tended to select some particular stimuli with a higher choice ratio and other stimuli with a lower choice ratio. In addition, the stimuli selected at a higher choice ratio and those selected at a lower choice ratio differed between monkeys.

### Relations Between the Choice Ratio and the Ratio of the Fixation Break During the Hold Period

**Figure 6:**
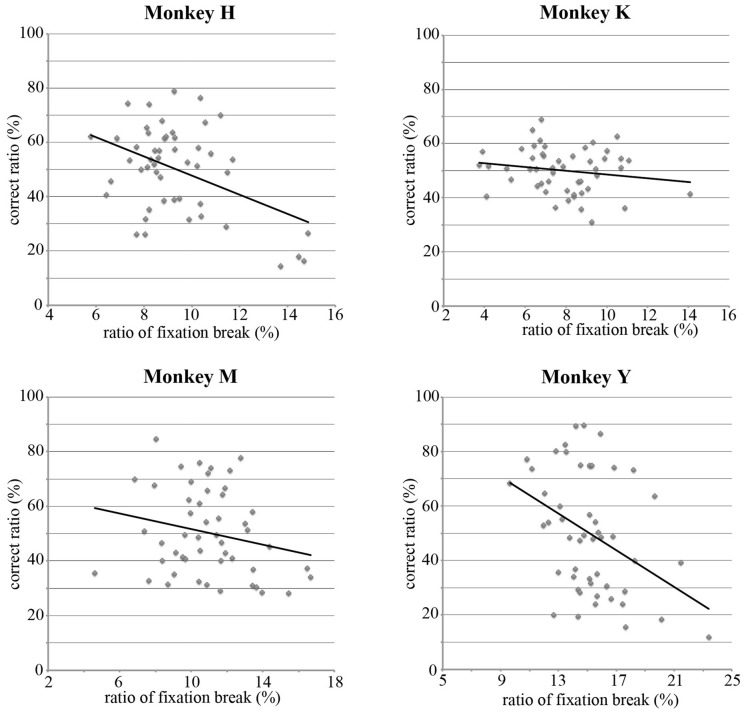
**Correlations between the choice ratio and the ratio of the fixation break during the hold period for each stimulus.** For four monkeys, the choice ratios were negatively correlated with the ratios of the fixation break (Monkey H, *r* = −0.448, *p* < 0.001; Monkey K, *r* = −0.172, *p* > 0.1; Monkey M, *r* = −0.219, *p* > 0.1; Monkey Y, *r* = −0.396, *p* < 0.005). Since monkeys were required to maintain looking at the selected stimulus for 3–6 s to obtain a reward, these results suggest that the choice ratios reflect the strength of the preference for visual stimuli.

In the present study, the monkey was rewarded only if it continued to look at the selected stimulus during the hold period. In the present study, since two visual stimuli were randomly selected from a pool of 50 stimuli, both selected stimuli were often those for which the monkey exhibited a lower choice ratio. When both stimuli selected by the computer had a lower choice ratio, the monkeys could take a strategy to use break fixation during the hold period to immediately finish the trial and expect to see the preferred stimulus in the next trial. If the monkey used this strategy, the choice ratio for each stimulus should be negatively correlated with the ratio of the break fixation during the hold period. This ratio was calculated for each of 50 stimuli by dividing the number of trials that the monkey broke fixation during the hold period by the total number of trials that the monkey selected this stimulus during the choice period. Figure 6 shows that, for all four monkeys, the choice ratios of the stimuli were negatively correlated with the ratios of the fixation break (Monkey H, *r* = −0.448, *p* < 0.001; Monkey K, *r* = −0.172, *p* > 0.1; Monkey M, *r* = −0.219, *p* > 0.1; Monkey Y, *r* = −0.396, *p* < 0.005). These results support the notion that the monkeys selected the stimulus during the choice period based on their preference for the stimulus.

### Physical Parameters of the Stimulus that Affected the Choice Ratio

#### Effect of Psychological Similarity Among the Stimuli

**Figure 7:**
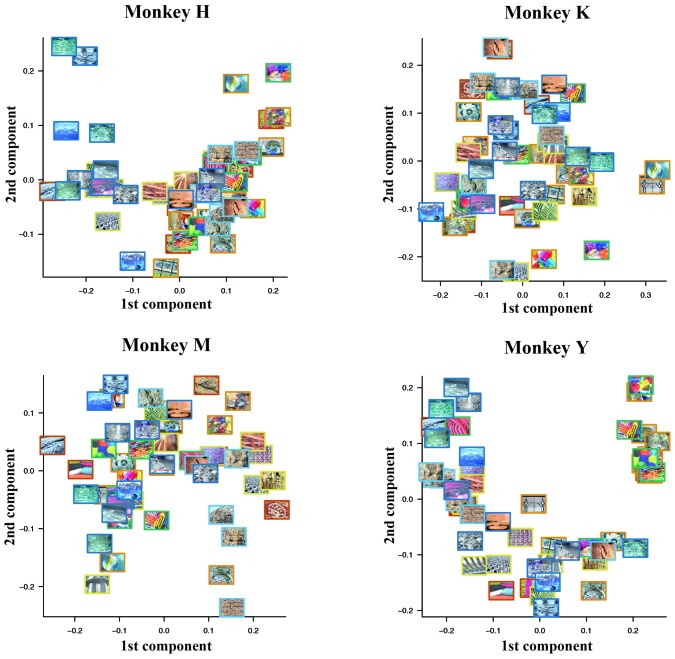
**Psychological similarity among the 50 visual stimuli calculated from the choice ratios of these stimuli.** We first calculated the psychological distance between two stimuli arbitrarily selected from the pool of 50 stimuli using the choice ratio of each stimulus. After we constructed a distance matrix using the obtained psychological distances among the 50 visual stimuli, we applied 2-D multi-dimensional scaling to this distance matrix. Monkey H seemed to classify the 50 stimuli into two groups based on their colorfulness and complexity; one group includes rather simple and monotone stimuli and the other group includes rather complex and colorful stimuli. A similar tendency for such classification was observed in Monkeys M and Y.

First, we examined whether or not the degree of psychological similarity among the 50 visual stimuli affected the pattern of the choice ratio. To assess the relation between the psychological similarity among the 50 visual stimuli and their choice ratios, we used a classical multidimensional scaling method (Torgerson, 1958; Mardia, 1978). For each monkey, we first calculated the psychological distance between two stimuli that were arbitrarily selected from a pool of 50 stimuli using the choice ratio of each stimulus. The psychological distance was defined as the difference of the choice ratio in these two stimuli from 0.5 (see “Materials and Methods” Section for details). We constructed a distance matrix using the obtained psychological distances among the 50 visual stimuli. Next, we applied a 2-D multi-dimensional scaling to this distance matrix. Figure 7 shows plots of the results. Monkey H seemed to classify the 50 stimuli into two groups based on their colorfulness and complexity; one group includes rather simple and monotone stimuli and the other group includes rather complex and colorful stimuli. A similar tendency for such classification was observed in Monkeys M and Y. However, we did not find similar tendency in Monkey K. This difference may be caused by the fact that Monkey K exhibited strong spatial bias in stimulus selection. Thus, the colorfulness and complexity of the stimuli might be important parameters for monkeys’ selection of visual stimuli.

#### Effect of Physical Properties of the Stimuli

To examine whether some physical property of the visual stimuli affected the monkeys’ selection of the stimuli, we calculated the average values of luminance, hue and saturation for each image and examined the correlations between the choice ratio and each of these physical parameters for each image. Table 1 summarizes the results of these analyses. Hue variance and saturation of the stimulus significantly affected the choice ratio of the stimuli in two monkeys (Monkeys H and Y), suggesting that colorfulness may be an important factor in determining the choice ratio of the stimuli for these monkeys. On the other hand, luminance and hue did not affect the choice ratio of the stimuli in any of the monkeys.

**Table 1:** **Relations between choice ratios and physical parameters of the stimuli**.

	Monkey H	Monkey K	Monkey M	Monkey Y
Choice ratio vs. Luminance	*r* = −0.01	*r* = −0.02	*r* = 0.15	*r* = 0.07
Choice ratio vs. Hue	*r* = −0.12	*r* = −0.02	*r* = −0.20	*r* = −0.08
Choice ratio vs. Hue variance	*r* = 0.45 (*p* < 0.01)	*r* = −0.01	*r* = −0.14	*r* = 0.52 (*p* < 0.01)
Choice ratio vs. Saturation	*r* = 0.31 (*p* = 0.03)	*r* = 0.16	*r* = −0.16	*r* = 0.33 (*p* = 0.02)

In the present study, we calculated the choice ratio for each stimulus by dividing the number of times that the monkey selected this stimulus and obtained a reward by the number of times that this stimulus was presented in the choice period. The choice ratio is an average number defined over all pairs in which a given stimulus was presented. Therefore, the choice ratio could be affected not only by the physical properties of that stimulus itself but also the properties of the stimulus that was paired and not selected. To examine whether the choice between a pair of the stimuli was affected by the difference of a particular physical feature between them, we calculated absolute difference of the choice ratio for each stimulus pair (1225 pairs) and absolute difference of physical parameters of these stimuli (luminance, hue, hue variance and saturation) and calculated correlations between these two values. Table 2 summarizes the results of this analysis. Significant effect was observed in the difference of the luminance between stimulus pairs for all monkeys. Although parameters related to color affected the choice ratio, the brightness of the stimulus was more important factor for all monkeys during the choice between a pair of the stimuli.

**Table 2:** **Correlation coefficients between the absolute difference of the choice ratio for each stimulus pair (1225 pairs) and the absolute difference of physical parameters (luminance, hue, hue variance and saturation) for each of these stimulus pairs**.

	Monkey H	Monkey K	Monkey M	Monkey Y
Luminance	−0.086** (*p* = 0.002)	0.116** (*p* = 0.000)	−0.073* (*p* = 0.011)	0.089** (*p* = 0.002)
Hue	0.032 (*p* = 0.263)	0.103** (*p* = 0.000)	0.031 (*p* = 0.277)	0.002 (*p* = 0.920)
Hue variance	0.063* (*p* = 0.028)	−0.052 (*p* = 0.071)	−0.033 (*p* = 0.247)	0.178** (*p* = 0.000)
Saturation	0.067* (*p* = 0.019)	0.045 (*p* = 0.118)	−0.025 (*p* = 0.378)	0.095** (*p* = 0.001)

*p < 0.05, **p < 0.01.

**Figure 8:**
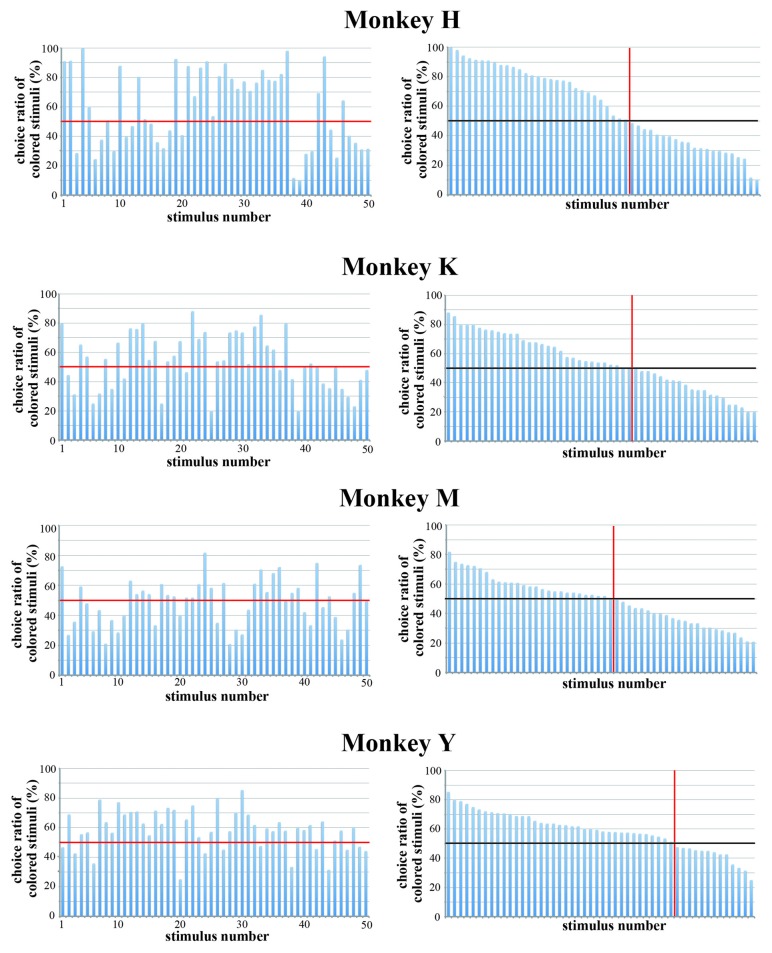
**Choice ratio of colored stimuli when the original color version and the black-and-white version of the same stimulus were presented simultaneously.** (Left) Choice ratios were arranged according to the stimulus number. (Right) Visual stimuli were arranged according to the rank order of their choice ratio. Red line indicates a choice ratio of 50%. The overall choice ratios of original color stimuli and black-and-white stimuli were 59.7% vs. 40.3% (paired *t*-test, *p* = 0.015) for Monkey H, 54.2% vs. 45.2% (paired *t-test*, *p* = 0.122) for Monkey K, 49.0% vs. 51.0% (paired *t-test*, *P* = 0.618) for Monkey M, and 58.5% vs. 41.5% (paired *t-test*, *P* < 0.01) for Monkey Y. The results indicate that the colorfulness of the stimuli is an important parameter for determining the monkey’s preference.

We also examined the monkeys’ choice behavior in trials in which an original color version and a black-and-white version of the same stimulus were presented simultaneously during the choice period. The stimulus was selected randomly from the pool of 50 stimuli. The monkeys were tasked to select either stimulus by eye movement. We performed this test for each monkey for five consecutive days and calculated the choice ratio. Figure 8 shows the choice ratio of colored stimuli. Monkeys H and Y, but not Monkeys K and M, exhibited a stronger preference for colored stimuli. We compared the choice ratio between an original color stimulus and a black-and-white stimulus for all 50 stimuli. The overall choice ratios of original color stimuli and black-and-white stimuli were 59.7% vs. 40.3% (paired *t*-test, *p* = 0.015) for Monkey H, 54.2% vs. 45.2% (paired *t-test*, *p* = 0.122) for Monkey K, 49.0% vs. 51.0% (paired *t-test*, *P* = 0.618) for Monkey M, and 58.5% vs. 41.5% (paired *t-test*, *P* < 0.01) for Monkey Y. Parameters related to color (hue variance and saturation) significantly affected the selection of the stimulus between a pair of the stimuli in Monkey H and Y (see Table 2). These results again indicate that the colorfulness of the stimuli is an important parameter for determining the monkey’s choice of stimuli.

#### Effects of the Complexity of the Stimuli

**Figure 9:**
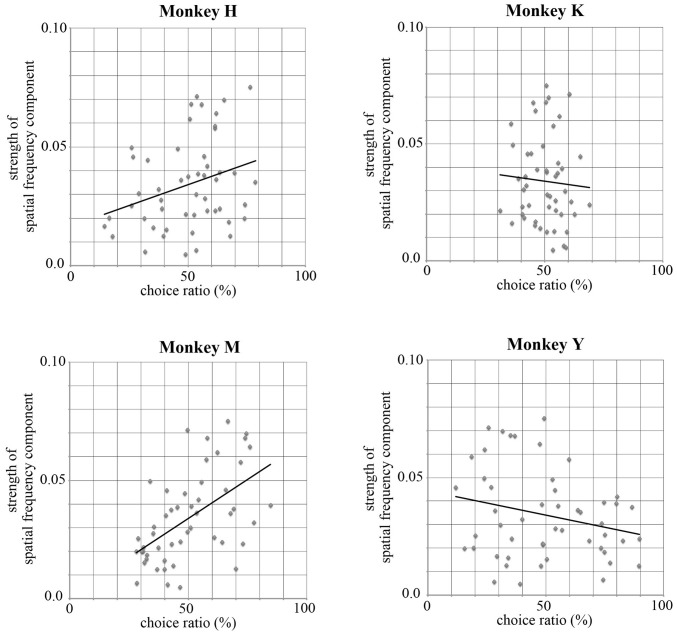
**Correlations between the statistical value for a high spatial frequency component with 0° orientation and the choice ratio of each stimulus.** Monkeys H and M showed a positive correlation (Monkey H, *r* = 0.298, *p* < 0.05; Monkey M, *r* = 0.550, *p* < 0.01), Monkey Y showed a negative correlation (Monkey Y, *r* = −0.241, *p* < 0.05), and Monkey K showed no correlation (Monkey K, *r* = −0.063, *p* > 0.1) between the choice ratio and the statistical value for a high spatial frequency component of the stimulus. Significantly correlation was observed for other orientations and for both high and middle spatial frequency components in Monkey M and Y. However, significant correlation was observed only in a few spatial components in Monkey H and was not observed in Monkey K. These results suggest that the monkeys’ selection of visual stimuli was affected by spatial frequency components included in the stimulus.

The complexity of the stimuli used in the present study can be estimated by the strength of their spatial frequency components. The strength of the spatial frequency component of each original image was calculated based on the method proposed by Portilla and Simoncelli (2000). All original images were transformed into monochrome images and the luminance of each monochrome image was decomposed into 12 sub-band images by applying a steerable wavelet pyramid transform. These sub-band images include three scales (high, middle, and low spatial frequencies) and four orientations (0°, 45°, 90° and 135°). We then obtained statistical values for the 12 sub-bands for each of the 50 images using software. These statistical values indexed the strength of particular spatial frequency components included in each of 12 sub-band images (e.g., strength of the high spatial frequency component at the 0° orientation). Next, we calculated the correlation coefficient between the strength of each spatial frequency component and the choice ratio of each stimulus. Figure 9 shows an example of this analysis using a value obtained for the high spatial frequency component with 0° orientation and the choice ratio of each stimulus. Two monkeys showed a positive correlation (Monkey H, *r* = 0.298, *p* < 0.05; Monkey M, *r* = 0.550, *p* < 0.01), one monkey showed a negative correlation (Monkey Y, *r* = −0.241, *p* < 0.05), and one monkey did not show any correlation (Monkey K, *r* = −0.063, *p* > 0.1) between the choice ratio and the statistical value of the high frequency spatial component of the stimulus used in the present study. As seen in Table 3, significantly positive and negative correlations were observed not only for the 0° orientation but also for other orientations and for both high and middle spatial frequency components in Monkey M and Y, indicating that spatial frequency components strongly affected stimulus selection in these two monkeys. However, significant correlations were observed only in a few spatial components in Monkey H, suggesting that this monkey’s preference was more affected by the colorfulness of the stimulus compared with spatial frequency components. On the other hand, significant correlation was not observed in any spatial component in Monkey K. This might be caused by strong spatial bias shown during the choice period. These results indicate that the monkeys’ selection was affected by spatial frequency components included in the stimulus.

**Table 3:** **Correlation coefficients obtained between choice ratio of each stimulus for each monkey and 12 sub-band components of each stimulus**.

	Monkey H	Monkey K	Monkey M	Monkey Y
High spatial frequency
0°	0.298*	−0.063	0.550**	−0.241*
45°	0.156	−0.020	0.567**	−0.351*
90°	−0.098	0.005	0.448**	−0.394**
135°	0.110	−0.031	0.606**	−0.358**
Middle spatial frequency
0°	0.312*	−0.168	0.572**	−0.173
45°	0.085	−0.119	0.585**	−0.299*
90°	−0.223	−0.046	0.388**	−0.351*
135°	0.062	−0.115	0.634**	−0.323*
Low spatial frequency
0°	0.239	0.018	0.438**	0.208
45°	−0.030	0.027	0.375**	0.099
90°	−0.340*	−0.119	0.251	−0.147
135°	−0.053	−0.068	0.531**	−0.038

*p < 0.05, **p < 0.01.

**Figure 10:**
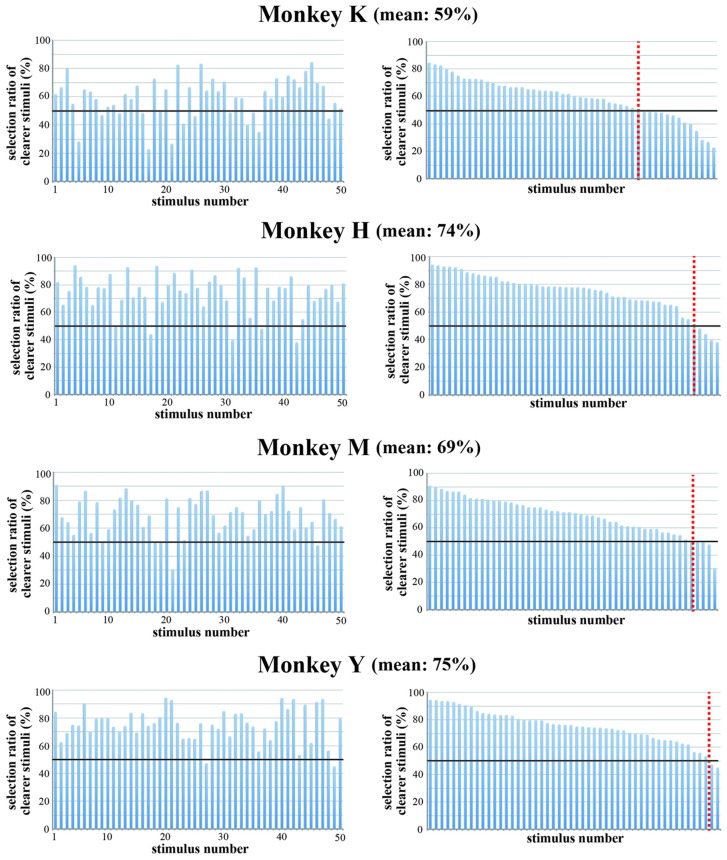
**Choice ratio of clearer stimuli when the original color version and the blurred version of the same stimulus were presented simultaneously.** (Left) Choice ratios were arranged according to the stimulus number. (Right) Visual stimuli were arranged according to the rank order of their choice ratio. Overall choice ratio of original clearer stimuli was 74.3% in Monkey H, 59.1% in Monkey K, 69.3% in Monkey M and 75.0% in Monkey Y. These results indicate that monkeys preferred to select distinct stimuli compared with blurred stimuli. Red line indicates a choice ratio of 50%.

To confirm that the strength of spatial frequency components affected the monkeys’ selection of visual stimuli, we made colored-blurred versions of stimuli from among the 50 original images and presented the colored-blurred version and the original colored version of the same stimulus simultaneously during the choice period. The stimulus presented during the choice period was selected randomly from the pool of 50 stimuli and the positions of the blurred stimulus and the original stimulus were randomized. All monkeys selected the colored original version more frequently than the colored-blurred version of the same stimulus (Figure 10). Overall, the choice ratio of original stimuli was 74.3% in Monkey H, 59.1% in Monkey K, 69.3% in Monkey M and 75.0% in Monkey Y. The number of stimuli for which the monkey selected the original version more often than the blurred version was 46 in Monkey H, 37 in Monkey K, 48 in Monkey M and 48 in Monkey Y. These results indicate that monkeys preferred to select complex and distinct stimuli compared with monotonous and blurred stimuli. Complex and distinct stimuli include a greater magnitude of spatial frequency components than monotonous and blurred stimuli. Therefore, monkeys more often selected stimuli with a greater magnitude of spatial frequency components.

## Discussion

### Monkeys Exhibit a Preference for Visual Images

An animal’s preference for stimuli or items has been examined using a simple choice task, in which the animal was requested to select one stimulus from among two or more different stimuli presented simultaneously, or by measuring how long or how many times the animal watched a particular stimulus. The strength of this preference can be estimated by the choice ratio for each stimulus, the duration or frequency that the animal watched each stimulus, or how close the animal comes to the stimulus. Studies on animals have revealed that animals exhibit a different preference for different stimuli.

In the present study, we used 50 photographs of a variety of items, materials and scenes and examined whether macaque monkeys exhibited preferences for these stimuli using a choice task. We found that each monkey exhibited different preferences for these stimuli, as shown in Figure 3. The observed preferences for the stimuli were not caused by a mere exposure effect (Zajonc, 1968, 2001), since: (1) two stimuli were randomly selected from the pool of 50 stimuli for each trial; (2) the total number of presentations was similar across the 50 stimuli; and (3) there was no significant correlation between the number of presentations and the choice ratio for each stimulus. In addition, monkeys obtained the same amount of reward independent of which stimulus they selected. Although monkeys exhibited individual differences in the preference of each of 50 visual stimuli, all monkeys exhibited similar preference for visual stimuli having similar physical properties. For example, all of the monkeys tended to more frequently select complex and colorful stimuli, as shown in Figures 3, 7. Also, they selected clear and in-focus stimuli, when a monotonic and in-focus stimulus was presented simultaneously with a colorful and out-of-focus image of the same stimulus. In addition, the most frequently selected stimuli and least frequently selected stimuli were maintained across many weeks of the experiments for all monkeys. These results indicate that the macaque monkeys’ preference observed in the present experiment is not caused by a mere exposure effect or a simple association between particular stimuli and a reward. Rather, the differences in the choice ratios of these stimuli are due to the differences in the monkeys’ preferences for these stimuli. Monkeys exhibit different degrees of preference for a variety of visual stimuli.

In our previous study (Takebayashi and Funahashi, 2009), we used artificially constructed fractal images, and found that macaque monkeys exhibited different choice ratios in response to different fractal stimuli. These monkeys showed individual differences in choice ratios, such that the pattern of choice ratios across fractal stimuli differed between monkeys. Thus, monkeys exhibited a preference for artificial visual stimuli. Current results agree with our previous results and indicate that macaque monkeys exhibit different strength of preference to a variety of visual images.

### Which Physical Features Determine a Monkey’s Preference for Visual Images?

Previous behavioral studies used rather simple graphic patterns or motion stimuli and indicated that macaque monkeys exhibit a preference for particular visual stimuli (Anderson et al., 2005; Blatter and Schultz, 2006). Takebayashi and Funahashi (2009) used artificial fractal stimuli and showed that monkeys exhibited different preferences for these colorful and complex stimuli. However, it is not known whether this difference in the preference for these stimuli depends on some difference in the physical features of the stimuli (e.g., colorfulness, brightness, complexity, spatial frequency) or which physical features determine the preference for the stimulus. Further, it is not known whether this preference is affected by the materials shown (e.g., fabric, paper, metal, plastic, glass and so on).

In the present study, we used 50 photographs obtained from the FMD. Since this database includes pictures of a variety of materials (fabric, foliage, glass, leather, metal, paper, plastic, stone, water and wood), it should be ideal for examining whether the materials photographed and their physical features affect the strength of the preference for visual stimuli. We selected 50 pictures (5 of fabric, 11 of glass, 9 of metal, 6 of plastic, 6 of stone and 13 of water) and examined the monkeys’ preferences for these stimuli. The results showed that the strength of monkeys’ preference was not related to the items and materials that were photographed, although, in general, the choice ratios of water and metal were low, while the choice ratios of glass and plastic were high compared with those of other materials. Most of the photographs of water, metal and stone were monotonic, while most of the photographs of fabric, glass and plastic were colorful and complex. Therefore, colorfulness and complexity would have stronger effect on the preference for visual stimuli. The evidence that the colorfulness of the stimulus would be one of the determinants of the preference was supported by comparison of the choice ratio between the original color version and the black-and-white version of the same stimulus. While two monkeys exhibited a stronger preference for colored stimuli, the other two monkeys did not show this preference. However, compared to the effect of colorfulness, the complexity and clarity of the stimulus were stronger determinants of the preference. All of the monkeys selected clear and distinct images more frequently than blurred images. These monkeys also more frequently selected images that included a greater amount of spatial frequency components. Thus, these results indicate that the physical features of the stimulus affect the strength of the monkey’s preference and the complexity, clarity and colorfulness of the stimulus are important determinants of the preference.

### What Determines the Preference for Visual Images?

Each animal species has particular stimuli that are intrinsically valuable and systematically attract its attention. For example, domestic chicks tend to choose a particular stimulus which was exposed during the imprinting phase (Bateson and Jaeckel, 1974) and exhibit ontogenetic pecking preference to a solid hemisphere over a flat disk (Dawkins, 1968). Since primates are highly visual and highly social animals, social cues such as conspecifics, faces, sex and social status are attractive stimuli and are important factors that determine the preference. It has been shown that social stimuli play important roles as discriminative stimuli to determine the preference (Anderson, 1998; Watson et al., 2012). Fujita (1987) showed that macaque monkeys visually discriminated and preferred to look at stationary images of their conspecifics. Méary et al. (2014) examined preference for faces between humans and rhesus monkeys and reported that rhesus monkeys preferred to see same species’ faces. Further, Swartz and Rosenblum (1980) used socially reared juvenile bonnet monkeys and showed the discrimination of their conspecifics among other macaque species and the preference to look at their conspecifics. The stimuli including social cues often act as a reinforcer for behavioral conditionings (Swartz and Rosenblum, 1980; Deaner et al., 2005; Watson et al., 2012). For example, Deaner et al. (2005) compared the strength of the reward value of photographs (female monkeys’ rumps or dominant male monkeys’ faces) or movies that monkeys preferred to watch with that of a food or liquid reward. They showed that some visual stimuli that reflect the social relationships of monkeys are associated with a stronger preference and reward value than a biological reward such as juice or food. Swartz and Rosenblum (1980) showed that a color movie indicating adult female bonnet monkeys moving freely in the experimental chamber could act as a reward to keep performing an operant conditioning task.

In the present study, most of the stimuli used were photographs of man-made artifacts and natural objects and no visual stimuli had any social meaning for monkeys. Therefore, it is concluded that the preference observed in the present study was not determined by the social importance of the stimuli. However, to recognize the faces of conspecifics, Gothard et al. (2009) showed that rhesus monkeys used both recognition of individuals with characteristic features of the face (feature-based processing) and recognition and discrimination of spatial difference of these features (holistic/configural processing). To recognize social cues such as individual faces, complexity, clarity and colorfulness are important features that those cues must have. Therefore, it is reasonable that complexity, clarity and colorfulness of the stimulus are important determinants of the preference for visual stimuli as was observed in the present study.

Another factor affecting monkey’s preference for visual stimuli is their novelty. It has been shown that the novelty affects preference for face stimuli in macaque monkeys (Gothard et al., 2004, 2009; Méary et al., 2014). Butler and Woolpy (1963) used a variety of visual stimuli and showed that rhesus monkeys preferred to view color motion pictures than stationary photographs. Other studies (Swartz and Rosenblum, 1980; Blatter and Schultz, 2006) also showed that motion pictures were more attractive stimuli among a variety of visual stimuli for monkeys. Butler and Woolpy (1963) suggested that stimulus change that occurred in motion pictures is an important factor for rhesus monkeys to determine the preference for visual stimuli. In addition, motion pictures continuously include new pictures and these pictures are presented in a coherent sequence. Coherent animation of motion pictures produces continuous change of visual stimuli. Therefore, continuous and coherent change of the stimuli could enhance the novelty of the stimuli. Since monkeys are attracted more to novel stimuli than familiar stimuli, the novelty produced by motion pictures might be an important factor to determine preference for motion pictures. In the present study, we used 50 stationary images and all these images were new for the monkeys at the beginning of the examination. Although a pair of the stimuli was selected randomly from a group of 50 images, different pair of images was presented in each different trial. However, the same group of images was used and repeatedly presented to the monkeys for 9–12 weeks. Therefore, it may not be the case that the preference for visual stimuli observed in the present study was affected by the novelty of the stimuli.

Pleasantness is also an important factor to determine the preference of stimuli. Humphrey (1972) proposed that rhesus monkey’s preference for visual stimuli could be explained by two independent factors; relative “pleasure” and relative “interest” of the stimulus. For example, when the monkey was asked to select one of single-colored plate between two, the monkey’s selection was not affected by novelty or familiarity or interest, but affected by relative pleasantness or unpleasantness (Humphrey, 1971). On the other hand, when the monkey was asked to select one picture between two, the monkey’s selection was affected more by novelty or familiarity or interest than relative pleasantness (Humphrey, 1971). He proposed that the strength of the “pleasure” factor could be determined by the difference of physical properties of the stimuli, whereas the strength of the “interest” factor could be determined by the difference of the contents that the stimulus had. Therefore, when monkeys continued to perform the task to select one stimulus between two, repetitive presentation of the same pair of stimuli could cause rapid fading of the “interest” factor, but stablilize the remaining of the “pleasure” factor.

In the present study, the rank order of the choice ratios for 50 stimuli was maintained across many weeks of experimental sessions for all monkeys, although the rank order of these stimuli fluctuated during the first or second week. In addition, the present results showed that the strength of monkeys’ preference was not related to the items and materials that were photographed. However, the physical features of the stimulus, such as complexity, clarity and colorfulness of the stimulus, affected the strength of the monkey’s preference. Based on the Humphrey’s hypothesis, the present results indicate that the preference observed in the present study was determined by the relative pleasantness or unpleasantness of the stimuli, not by the relative “interest” of the stimulus.

Recently, the relations between the preference for paintings and photographs and brain structures have been examined using human subjects by asking to rate these images with respect to subject’s preference during brain scans (McWhinnie, 1968; Aharon et al., 2001; Kawabata and Zeki, 2004; McClure et al., 2004; Vartanian and Goel, 2004a,b; Nadal et al., 2008). These studies demonstrated that activation was observed in the nucleus accumbens and the orbitofrontal cortex which are related to positive emotion like pleasantness and reward, and that the magnitude of activation in these brain areas co-varied as a function of the preference ratings (Aharon et al., 2001; Kawabata and Zeki, 2004; Vartanian and Goel, 2004b). However, no physiological experiment has been performed to examine how the preference of a variety of visual stimuli is determined, which brain areas participate in preference judgment, and how preference judgment is performed in these brain areas using monkeys. Further studies are needed to examine these issues.

## Author Contributions

SF performed whole experiment, analysis and writing of the article.

## Conflict of Interest Statement

The author declares that the research was conducted in the absence of any commercial or financial relationships that could be construed as a potential conflict of interest.

## Footnotes

^1^http://people.csail.mit.edu/celiu/CVPR2010/FMD/
